# Severe anaemia is not associated with HIV-1 *env *gene characteristics in Malawian children

**DOI:** 10.1186/1471-2334-8-26

**Published:** 2008-02-29

**Authors:** Job CJ Calis, Hellen P Rotteveel, Antoinette C van der Kuyl, Fokla Zorgdrager, David Kachala, Michaël Boele van Hensbroek, Marion Cornelissen

**Affiliations:** 1 Emma Children's Hospital, Academic Medical Centre, Amsterdam, The Netherlands; 2 Malawi-Liverpool-Wellcome Trust Clinical Research Programme, College of Medicine, Blantyre, Malawi; 3 Laboratory of Experimental Virology, Department of Medical Microbiology, Centre for Infection and Immunity Amsterdam (CINIMA), Academic Medical Centre of the University of Amsterdam, Amsterdam, The Netherlands; 4 Liverpool School of Tropical Medicine, Liverpool, UK

## Abstract

**Background:**

Anaemia is the most common haematological complication of HIV and associated with a high morbidity and a poor prognosis. The pathogenesis of HIV-associated anaemia is poorly understood and may include a direct effect of HIV on erythropoiesis. *In vitro *studies have suggested that specific HIV strains, like X4 that uses the CXCR4 co-receptor present on erythroid precursors, are associated with diminished erythropoiesis. This co-receptor affinity is determined by changes in the hypervariable loop of the HIV-1 envelope genome. In a previous case-control study we observed an association between HIV and severe anaemia in Malawian children that could not be fully explained by secondary infections and micronutrient deficiencies alone. We therefore explored the possibility that alterations in the V1-V2-V3 fragment of HIV-1 were associated with severe anaemia.

**Methods:**

Using peripheral blood nucleic acid isolates of HIV-infected children identified in the previous studied we assessed if variability of the V1-V2-V3 region of HIV and the occurrence of X4 strains were more common in HIV-infected children with (cases, n = 29) and without severe anaemia (controls, n = 30). For 15 cases bone marrow isolates were available to compare against peripheral blood. All children were followed for 18 months after recruitment.

**Results:**

Phylogenetic analysis showed that HIV-1 subtype C was present in all but one child. All V1-V2-V3 characteristics tested: V3 charge, V1-V2 length and potential glycosylation sites, were not found to be different between cases and controls. Using a computer model (C-PSSM) four children (7.8%) were identified to have an X4 strain. This prevalence was not different between study groups (p = 1.00). The V3 loop characteristics for bone marrow and peripheral blood isolates in the case group were identical. None of the children identified as having an X4 strain developed a (new) episode of severe anaemia during follow up.

**Conclusion:**

The prevalence of X4 strains in these young HIV-1-subtype-C-infected children that were most likely vertically infected and naïve to anti-retroviral therapy can be considered high compared to previous results from Malawi. It is unlikely that V1-V2-V3 fragment characteristics and HIV co-receptor affinity is an important feature in the development of severe anaemia in Malawian children.

## Background

Anaemia is the most common haematological complication of HIV in adults and children worldwide [1-3] and is associated with a reduced quality of life and a high morbidity [4]. Inadequate erythropoiesis is generally considered to be the main pathophysiological mechanism of HIV-associated anaemia [1,5,6]. Despite the obvious medical importance of anaemia the aetiology of this erythropoietic failure is still not well understood. Several possible pathways have been investigated including opportunistic infections, micronutrient deficiencies and the more recently identified direct effect of HIV on erythropoiesis [3,7]. The induction of anaemia by specific strains of HIV is an example of this direct of effect of HIV [8].

Early in infection the HIV-1 population usually consist of a strain that has the capacity to bind to both CD4 and the co-receptor CCR5 (R5 strain)[9,10]. Later in infection a broadening or switch occurs and HIV evolves to infect cells expressing CD4 and the co-receptor CXCR4 (R4 strains) [10,11]. This switch is thought to occur in 50% of infections and is associated with an accelerated loss of CD4+ T-cells and progression to AIDS [12].

Like the T-helper cells, erythropoietic stem cells express both CD4 and CXCR4 on their membrane[13,14]. Although productive infection is uncommon in erythroid precursor cells [3], several *in vitro *studies have associated X4 strains with cell death in erythroid and other cell lines [7,15-17]. Another similarity between T-helper cells and erythroid cells is the decline in both cell types during disease progression [3,7]. Large studies have suggested that anaemia might even be a better predictor of mortality than loss of CD4 cells or HIV load increase [5,18,19]. A decline of 1 g/dL in the haemoglobin concentration was associated with a greater increased hazard of death than a halving of the absolute CD4 count or a log increase in viral load [18]. The reversal of anaemia, again similar to an increase in T-helper cells, was associated with a better life expectancy [18,19]. Despite these similarities to T cells, no study has evaluated if the decrease in erythrocytes might be a direct or indirect consequence of an alternated co-receptor affinity [16].

Co-receptor affinity is a consequence of changes in the variable loops (V1-V2-V3) of the envelope protein (*env*) of HIV-1. Especially a high V3 amino acid charge was found to be associated to X4 co-receptor affinity [20-24] and several models have been published to predict co-receptor affinity using V3 data [25-28]. Changes to the V1-V2 fragment appear to be more indirectly linked to X4 affinity and are increasingly associated with a neutralizing antibody escape [29-37]. These changes include an increased number of potential N-linked glycosylation sites in V1-V3 [30-35] and possibly an extended length of the (V1-)V2 fragment [29,36,37].

In a recent case-control study we found a strong association between HIV and severe anaemia in African children [38]. This association could partly be explained by opportunistic infections with micronutrient deficiencies playing only a modest role. After correction for this an independent association remained which can be explained by a direct effect of HIV on erythropoiesis. We hypothesized that the occurrence of X4 strains and other alterations of the hypervariable loops V1-V2-V3 of the HIV-1 *env *genome would be associated with severe anaemia. Using nucleic acid isolates of HIV-infected children with (cases) and without (controls) severe anaemia we assessed the prevalence of: (1) high V3 and total V1-V2-V3 amino acid charge; (2) an extended V1-V2 length; (3) an increase of potential N-linked glycosylation sites of the V1-V3 and V3 fragments; (4) a HIV-1 subtype C position specific scoring matrix (C-PSSM) that predicts co-receptor usage[25].

## Methods

In two hospitals in southern Malawi we recruited two groups of children aged 6–60 months into a case-control study on severe anaemia (Haemoglobin concentration < 5 g/dL) as previously described [38]. In short, a severely anaemic child requiring a blood transfusion (*Case*, Haemoglobin concentration < 5 g/dL) was recruited at presentation to hospital alongside two controls (Haemoglobin concentration ≥ 5 g/dL). One control was recruited from apparently healthy residents living within proximity of the case-patient (*Community Control*, *CC*) the other randomly selected at the outpatient department (*Hospital Control, HC*). On enrolment, a standardized study questionnaire and physical examination were completed, and blood samples were collected. In cases only, a bone marrow aspiration was performed under anaesthesia if the clinical condition permitted. Nutritional Z-scores were calculated in EPI info 2000 [39]. 'Wasting' (weight-for-height), applied to children with Z-scores <-2. Children requiring admission were treated in a study ward. All conditions were managed according to standard protocols. All three study groups (cases, HC and CC) were actively followed at 1, 3, 6, 12 and 18 months. In addition, children were passively followed by asking guardians to return to study clinics whenever the child was sick. During follow-up visits clinical data was collected and a peripheral blood haemoglobin concentration was determined. Fully informed consent was obtained from a parent or guardian in all three study groups. HIV testing was discussed after transfusion for the cases and on a follow-up visit for controls. The study was approved by the ethics committees of the College of Medicine, Malawi, and the Liverpool School of Tropical Medicine, UK.

### Laboratory tests on site

Haemoglobin was measured on site using a Hemocue system (Angelholm, Sweden). A full blood and reticulocyte count was performed by Coulter counter (Coulter, Hialeah, Fla).

C-reactive protein (CRP) was analyzed in heparin plasma on a Roche p800/p170 system (Roche, Switzerland). HIV testing was performed using two rapid tests (Determine, Abbott-Laboratories, Japan; Unigold, Trinity-Biotech, Ireland). Reactive results in children less than 18 months and discordant outcomes were resolved by PCR. Lymphocyte subsets including CD4 cell counts were measured, during the second year of recruitment, by adding 50 μl of whole blood to TRUECOUNT absolute count tubes (Becton Dickinson, USA) and incubated with 20 μl MultiTest reagent. After incubation and red cell lysis, the cells were analysed on a Becton Dickinson FACSCalibur flow cytometer and analysed using MultiSet software (Becton Dickinson, USA). CD4 expressing T-cells were expressed as percentage of the total lymphocyte population and age adjusted cut-offs were used to define immunodeficiency[40]. Peripheral blood samples were separated and aliquots of serum and plasma were stored at -80C for later testing.

### DNA extraction and polymerase chain reaction (PCR)

**Figure 1:**
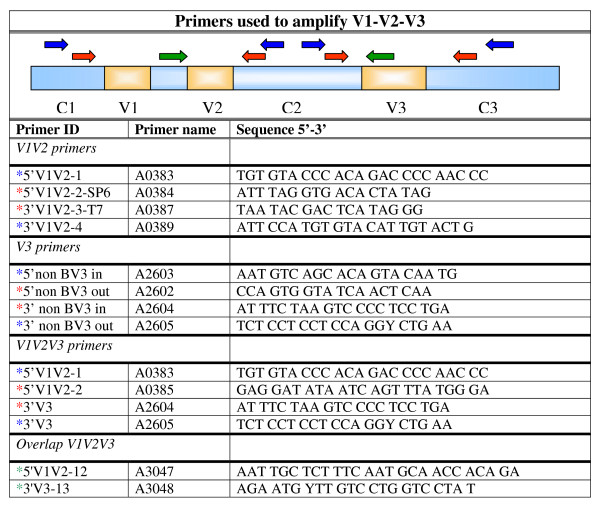
**PCR Primers used to amplify V1-V2-V3 fragment**. A display of the primer sequences used to amplify the V1-V3 fragment located on the viral genome of the HIV-1 *env *protein. External PCR: primers indicated with a blue *. Internal PCR primers are indicated with a red * for the V regions and with a green * for the C regions.

DNA was isolated from the blood and bone marrow samples with a silica-guanidiniumthiocyanate based method[41]. The target DNA (*env *gene) was amplified using polymerase chain reaction (PCR) with a pair of target-specific sense and antisense primers (Figure 1) in a 96-well 9700 thermocycler (Applied Biosystems, USA). First a reverse transcriptase (RT)-PCR was performed using 10 μl of the eluated nucleic acid solution and Avian Myeloblastosis Virus reverse transcriptase (AMV-RT, Boehringer Mannheim) to generate cDNA. Five microliter of the PCR product was used with Amplitaq (Applied Biosystems, USA) to amplify the V1-V2-V3 *env *(810 bp) gene fragment, or in case this failed a combination of nested PCRs was performed to amplify this region as fragments (primers Figure 1).

### Sequencing and cloning

PCR products were directly sequenced with the ABI Prism Big-dye Terminator v 1.1 Cycle Sequencing Kit in an ABI Prism 377 DNA sequencer using multiple fluorescent dyes (Applied Biosystems, USA). PCR fragments showing evidence of recombinants or a dual infection were cloned with TA TOPO cloning kit (Invitrogen, USA). For each sample at least eight clones were sequenced and from these a consensus sequence was made. All sequences were manually analysed and assembled with CodonCode Aligner (version 1.5.2.). Multiple sequences were aligned with CLUSTAL W [42] and optimised manually with BioEdit Sequence Alignment Editor (version 7.0.1).

### Viral Load determination

The HIV-1 viral load in plasma was determined with an in-house real-time PCR assay, with primers located in the HIV-1 pol gene. Primer/probe sequences were: upstream primer 5'TGC ATT YAC CATACC TAG T 3', downstream primer 5'ATT GCT GGT GAT CCT TTC CA 3', and probe 5'AAA CAA TGA GAC ACC AGG GAT TAG ATA 3'. The probe was 6-FAM labelled. The detection limit of this assay was 5 HIV-1 RNA copies per reaction.

### Phylogenetic analysis

Genetic subtypes were determined by phylogenetic analysis. The *env *gene sequence fragments were aligned with the corresponding reference sequences obtained from the Los Alamos HIV sequence database [43]. Phylogenetic reconstruction was carried out with Molecular Evolutionary Genetics Analysis (MEGA) software version 3.0. A model based upon the Kimura two-parameter model with pair wise deletion was used. Bootstrap values were based on a generation of 1000 replicate trees. Newly found recombinant forms were analysed using Simplot (version 3.5.1.0.) comparing recombinant sequences against a background of reference sequences.

### Potential N-linked glycosylation sites and Amino acid charges

All protein fragments containing Asparagine (N) and Serine (S) or Threonine (T) that were separated by any third amino acid (X) other than Proline (P) were counted as a glycosylation site (N-X-S/T) [44]. All possible glycosylation sites and the amino acid charges were counted for the V3 and V1-V2-V3 fragment separately.

### Subtype C-specific phenotype predictor (C-PSSM)

A C-PSSM predictor is available online [45]. This predictor is based on the computational techniques presented in the paper from Jensen and colleagues [25].

### Statistics

We compared characteristics of HIV-infected cases to HIV-infected controls using the Fisher exact and Chi-square test (categorical data) and the t-test or Wilcoxon rank sum test (continuous data). For 2 × N contingency tables the Fisher-Freeman-Halton exact test was used. For all tests a two-sided alpha of <0.05 was used to assess significance. The tested sample size would be able to detect an odds ratio of 8 or more assuming an X4 strain prevalence of 8% in the control population, a power of 80% and an alpha of 0.05. Analyses were performed using SPSS 12.0 (SPSS inc, USA) and StatsDirect 2.6 (StatsDirect ltd, UK).

## Results

### Patient Characteristics

The current study was embedded into a larger case-control study that followed children for an additional 18 months. That study recruited 381 children with severe and 757 children without severe anaemia over a two year period. Overall 1039 (91%) parents consented to HIV testing, 57 (5.0%) refused and 42 (3.7%) were lost to follow up before counselling. Of all children tested 45 (13%) of severely anaemic cases and 41 (6%) non-severely anaemic controls were HIV-infected (p < 0.001). These 86 children formed the current study population and their baseline characteristics are described in Tables 1 and 2. Haemoglobin levels were lower in cases than controls but reticulocyte counts were not different between the study groups (p = 0.72). Of all HIV-infected children, cases were more likely to have been admitted to hospital in the past, were more wasted, immunodeficient for age and had higher plasma viral loads. Though the latter two results were only tested in a subset of children, none of these differences reached significance (Table 2). During follow-up, HIV-infected children with severe anaemia had a significant increased mortality (53%) compared to those without severe anaemia (15%).

**Table 1:** Characteristics of the HIV-infected children per study group.

	**Cases**	**Controls**	**p**
HIV-infected	45	41	n/a
Age (mean in months ± SD)	25.5 ± 14.1	28.8 ± 12.4	0.25
Sex (M:F)	18:27	23:18	0.14
Haemoglobin (mean in g/dL ± SD)	3.6 ± 0.7	9.3 ± 2.0	n/a
Reticulocytes (Median and IQR *10e9/L)	58.6 (30.3–93.1) n = 35	55.7 (34.7–86.0) n = 31	0.72
CRP>10 mg/L	38/42 (90%)	25/36 (69%)	0.02

Baseline characteristics of children with severe anaemia (cases, Hb<5.0 g/dL) as compared to those without severe anaemia (controls). SD: Standard deviation; IQR Inter Quartile Range; The number of children is only displayed if data was not available for all children.

**Table 2:** Markers of disease progression and outcome study group.

	**Cases**	**Controls**	**p**
Wasting (Z-score weight for height <-2)	7/36 (19%)	5/38 (13%)	0.46
Previous hospital admission	25/44 (57%)	16/41 (39%)	0.10
Mortality (18 months)	27/45 (60%)	6/41 (15%)	<0.001
During admission	7/45 (16%)	0/41 (0%)	0.01
After discharge	20/38 (53%)	6/41 (15%)	<0.001
Plasma viral load (mean ± SD in Log10/mL)	6.02 ± 1.08 n = 13	5.43 ± 0.85 n = 12	0.15
Severe immunodeficient (based on CD4%*)	9/16 (56%)	5/12 (42%)	0.45

Markers of disease progression in children with severe anaemia (cases Hb<5.0 g/dL) as compared to those without severe anaemia (controls). SD: Standard deviation; Viral loads were performed using a in-house methods as described in the method section. *According to the revised WHO guidelines CD4 <25% (6–12. months); <20% (12–35 months); <25% (36–59 months) [40].

### Sample Processing

Overall 29 (64%) of HIV-infected cases and 30 (73%) of HIV-infected controls had samples available for further analysis of the *env *fragment (Table 3). For one patient the PCR failed, resulting in a recovery rate of 98%. For seven other samples the *env *fragment could only be partially amplified (V3 failed: n = 5 and C2 failed: n = 2). Cloning was needed to determine consensus sequences for 14 samples. In one sample V1-V2 fragment the nucleotide differences were too large between clones, two consensus sequences were made and separately analysed. However V3 loops of these clones were found to be identical. Degenerate base counts were taken into account and were not different between cases and controls (p = 0.33, Table 3).

**Table 3:** Variability and predictors of co-receptor affinity per study group.

	**Cases**	**Controls**	**p**
Available for testing	29/45 (64%)	30/41(73%)	0.38
PCR failed	0/29	1/30	1.00
Complete *env *product	25/29	26/29	1.00
Degenerate base counts (median, range)	2 (0–21)	4 (0–36)	0.33
Viral type C	29/29	28/29	1.00
V3 amino acid charge ≥ +5*	0/25	4/27	0.11
X4 strains (C-PSSM)*	2/25	2/27	1.00

Presence of several indicators of co-receptor affinity in children with severe anaemia (cases, Hb<5.0 g/dL) as compared to those without severe anaemia (controls). * according to C-PSSM score by Jensen [25], available at [45].

### Phylogenetic analysis

**Figure 2:**
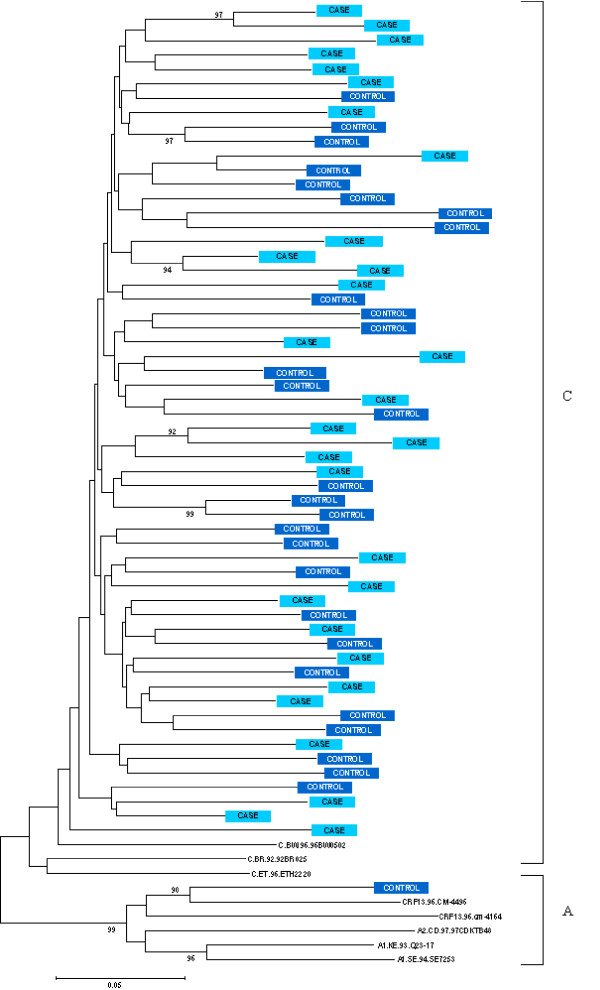
**Phylogenetic tree based on the V1-V2 fragment of the HIV-1 *env *gene nucleotide sequences**. Phylogenetic tree to assess if the HIV-1 genome of children with severe anaemia (cases, Hb<5.0 g/dL) is different from those without severe anaemia (controls). Subtype A and CRF-13 reference sequences are used as outgroup sequence.

Phylogenetic analysis of V1-V2 nucleotide sequences showed that 57 of 58 isolates clustered with subtype C reference sequences (bootstrap value: 99%, Figure 2). Only one child, a child recruited as control, had a new circulating recombinant form that showed similarity with CRF13-cpx, which has genomic regions identified as subtypes A, G, and J [46].

### Amino acid charges V fragments

**Figure 3:**
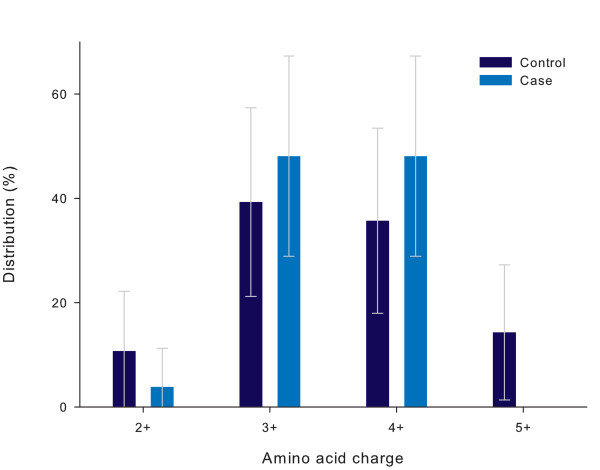
**Distribution of V3 amino acid charges per study group**. Distribution of the nett charges of the V3 fragment in children with severe anaemia (cases, Hb<5.0 g/dL) as compared to those without severe anaemia (controls). The distribution is expressed as a percentage of the total number of codons analysed per study group (Controls: n = 28, Cases n = 26). Error bars express 95% Confidence intervals. p = 0.19.

**Figure 4:**
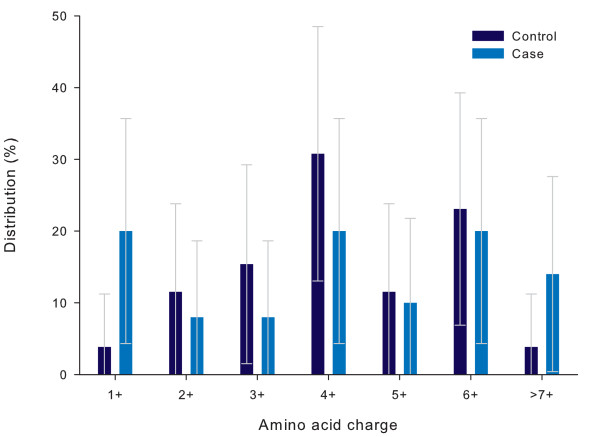
**Distribution of total amino acid charge of the V1-V2-V3 fragment per study group**. Distribution of the nett charges of the V1-V2-V3 fragment in children with severe anaemia (cases, Hb<5.0 g/dL) as compared to those without severe anaemia (controls). The distribution is expressed as a percentage of the total number of fragments analysed per study group (Controls: n = 26, Cases n = 25). Error bars express 95% Confidence intervals. p = 0.36.

Amino-acid charges of the V3 loop and the total V1-V3 fragment are displayed in Figure 3 and 4 respectively. The charges of the subtype C isolates ranged from +2 to +5 (V3) and from 1 to +7 (V1-V3) and were not significantly different between the study groups (p = 0.19 and 0.36 respectively). Higher V3 charges (+5 or more) were found in four isolates tested (7.7%), all in controls (Table 3). Additional file 1 shows the amino acid logos for the V3 loop per study group. Alterations of the amino acid in positions, 5–9, 11 and 25 of the V3 loop were not different (p > 0.2 for all).

### Potential N-linked glycosylation sites

**Figure 5:**
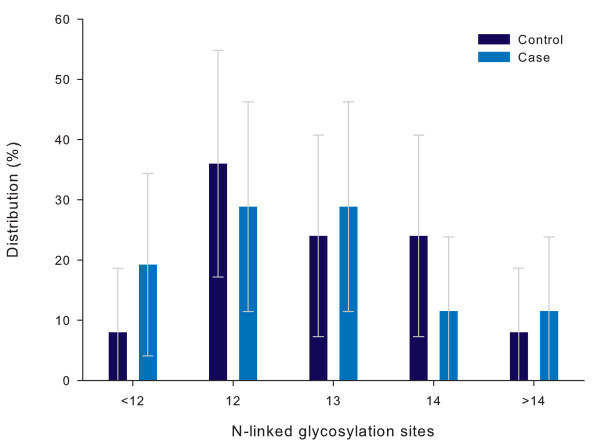
**Distribution of potential N-linked glycosylation sites on V1-V2-V3 per study group**. Distribution of number of potential N-linked glycosylation sites on the V1-V2-V3 fragment in children with severe anaemia (cases, Hb<5.0 g/dL) as compared to those without severe anaemia (controls). The distribution is expressed as a percentage of the total number of fragments analysed per study group (Controls: n = 25, Cases n = 26). Error bars express 95%. p = 0.75.

All isolates contained one potential N-linked glycosylation site in the V3 loop. The number of potential glycosylation sites on the V1-V3 fragment ranged between 10 and 17 (Figure 5) and was not different amongst cases and controls (p = 0.75).

### Length of the V1-V2 fragment

**Figure 6:**
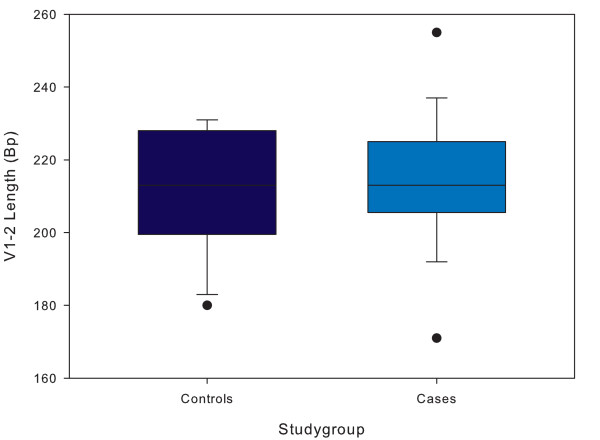
**Boxplots of V1-V2 length per study group**. Distribution of number of length of the V1-V2 fragment in children with severe anaemia (cases, Hb<5.0 g/dL) as compared to those without severe anaemia (controls). The length is expressed as in basepairs (bp). The analysis included 29 controls and 29 cases. Error bars express 95% confidence intervals, outliers are presented by dots. p = 0.55

The distribution of the length of the V1-V2 fragment ranged from 177 to 255 base pairs and is displayed in Figure 6. Mean length for cases was not significantly different from controls (214 vs. 211 base pairs respectively, p = 0.55).

### C-Position Specific Scoring Matrix

C-PSSM scores were used to predict co-receptor affinity for HIV-1 subtype C strains using V3 loop data. Within our study X4 tropism was found in 2 of 25 (8.0%) cases and 2 of 27 controls tested (7.4%, p = 1.0, Table 3). Both C-PSSM results for the child with two different clones suggested identical co-receptor affinity (CCR5). One of four children identified with an X4 strain according to this method had previously received a blood transfusion.

### Bone Marrow isolates

Some studies have suggested different compartments of the body may contain different viral strains. For 15 cases bone marrow samples were available. Synonymous changes in the amino acid sequences of the V3 fragments between bone marrow and peripheral blood isolates were observed in three children (20%). These patients showed alterations of amino acid structure at position 25 (n = 2, both e->d), and 29 (n = 1, n->d), changes that did not effect the C-PSSM prediction.

### Follow-up

After recruitment all children were followed during an 18 month follow-up period as part of this study. During this follow-up period none of the four children (two cases and two controls) that were infected with an R4 virus were diagnosed with a (new) episode of severe anaemia. The two cases died during follow up at 65 and 182 days from recruitment from other causes than severe anaemia.

## Discussion

Severe anaemia is major cause of morbidity and mortality in African children [47]. We previously reported that in an urban environment as many as 21% of children admitted with severe anaemia were HIV-infected which was more than twice as prevalent as for the control population. This association could only partly be explained by more prevalent secondary infections and an additional modest contribution of nutrient deficiencies. A direct effect of HIV on erythropoiesis was suspected and we assessed if HIV-1 envelope characteristics and co-receptor affinity were associated with the occurrence of severe anaemia in these children. In this first study to assess this association using a case-control design, we found no variations in the genetic domain of HIV *env *or the predicted prevalence of X4 strains between children with and without severe anaemia.

HIV-1 subtype C appeared to be the most prevalent subtype in our sample. Previous reports on adults and children in the region have confirmed that in Malawi HIV-1 subtype C is the most prevalent variant [48,49]. A new recombinant form was identified in one of the control children and showed similarities to CRF13-cpx, recently found in Cameroon [50]. Since this strain is very different from all other isolates found in Malawi, it might have spread from Central Africa by the major transports route running through the rural area in which this was found.

The phylogenetic tree based on the data from the *env *protein did not show clustering of cases as compared to controls and argues against our hypothesis that specific strains of HIV would predispose to the development of severe anaemia. More specifically we assessed V3 amino acid charge, overall V1-V2-V3 fragment charge, potential N-linked glycosylation sites on the V1-V2-V3 and the V3 fragments, and V1-V2 length and did not identify any significant difference between HIV-infected children with and without severe anaemia.

Although much has been published on co-receptor affinity for HIV-1 subtype B, the strain commonly found in western settings, relatively little is known on HIV-1 subtype C and co-receptor affiliation. Recently Jensen et al. published a validated algorithm to predict co-receptor usage in subtype C [25]. This C-PSSM score predicted X4 affinity in four strains, two in each study group.

The X4-strain prevalence of 7.7% in the case and control groups combined was higher than expected in this predominantly HIV-1 subtype C infected population [20,48,51,52]. Both studies reporting on co-receptor usage in Malawian populations did not identify a single X4-strain in both an adult [52] and paediatric [48] population. Others have published higher prevalences of X4-strains in HIV-1 subtype C-infected patients, however these reports came from Zimbabwe and South Africa and concerned adults in end-stage HIV infection (17–36%) [20,51]. Furthermore, this prevalence is remarkable since most children will have been vertically infected which is commonly considered to occur by R5 variants [9]. Previous studies have suggested that the occurrence of X4 strains in subtype C infections was associated with the use of anti-retroviral therapy (ART) [51]. No child was receiving ART at the time of this study.

The link between the occurrence of an X4 strain and disease progression is well studied in HIV-1 subtype B-infected persons. Little data is available concerning this association in HIV-1 subtype C-infected individuals. Our results suggest X4 variants are not uncommon in a HIV-1 subtype C-infected population and their survival, especially in the presence of anaemia, may be limited. Therefore more attention should be given to the clinical importance of the occurrence of this variant in HIV-1 subtype C infected children and adults.

A limitation of our study is that we used indirect measures to define co-receptor affinity rather than to assess actual infectivity of cell-lined expressing CXCR4 or CCR5. Since the C-PSSM method applied had a 94% specificity and 75% sensitivity [25], we might have underestimated X4 tropism in our entire population. Since this underestimation would have affected both our cases and controls it is unlikely to have had a major impact effect on our findings. We therefore did not pursue assessing our hypothesis using the more costly and laborious exercise of infecting cell lines expressing CXCR4 and CCR5. Although our data cannot fully refute an association between X4 strains and severe anaemia, X4 tropism is not a major cause of severe anaemia.

The study had several strengths including the case-control design and the long term follow-up period. This allowed a cross-sectional analysis of X4 infected children with a longitudinal assessment. We hypothesized that the occurrence of an X4 strain would predispose to severe anaemia. The cross-sectional design used may have been underpowered to detect a difference finding only four X4-infected children. However none of these four children developed a new episode of severe anaemia in the longitudinal study. This may be considered additional evidence against our hypothesis. Another strong point of the study was the availability of bone marrow samples in a subgroup of patients. The similarity of these isolates to those obtained from the peripheral blood argues against compartmentalisation in the bone marrow and strengthens our findings.

## Conclusion

In summary, we assessed whether HIV-1 *env *characteristics and CXCR4 co-receptor affinity were associated with the occurrence of severe anaemia in Malawian children. In this first study assessing clinical relevance we were unable to find any differences either by phylogenetic analysis and several tests used to assess co-receptor usage. We identified a relatively high prevalence of X4 strains in these HIV-1 subtype C-infected children that were young, most likely vertically infected and naïve to anti-retroviral therapy. More attention should be given to the clinical importance of the occurrence of this variant in HIV-1 subtype C infected children and adults.

## Competing interests

The author(s) declare that they have no competing interests.

## Authors' contributions

JC conceived the study, was responsible for the on site laboratory work, statistical analysis and drafted the manuscript. HPR carried out the PCR and sequence analysis, and helped to draft the manuscript. DK was responsible for design and performance of the on site laboratory tests and reviewed the manuscript. MBvH helped conceiving the study and drafting the manuscript. ACvdK, FZ and MC participated in the design and coordination of the study and contributed to the laboratory work. All authors read and approved the final manuscript.

## Pre-publication history

The pre-publication history for this paper can be accessed here:



## Supplementary Material

Additional file 1 **Frequency amino acid codon logos of the V3 loop per study group**. Sequence logos of HIV-1 V3 sequences found in children with severe anaemia (cases, Hb<5.0 g/dL, n = 25) as compared to those without severe anaemia (controls n = 28). The character and size of each logo represent the amino acid and its prevalence at the specific site. Click here for file
